# Common Carotid Intima Media Thickness and Ankle-Brachial Pressure Index Correlate with Local but Not Global Atheroma Burden: A Cross Sectional Study Using Whole Body Magnetic Resonance Angiography

**DOI:** 10.1371/journal.pone.0099190

**Published:** 2014-06-16

**Authors:** Jonathan R. Weir-McCall, Faisel Khan, Matthew A. Lambert, Carly L. Adamson, Michael Gardner, Stephen J. Gandy, Prasad Guntur Ramkumar, Jill J. F. Belch, Allan D. Struthers, Petra Rauchhaus, Andrew D. Morris, J. Graeme Houston

**Affiliations:** 1 Division of Cardiovascular and Diabetes Medicine, Medical Research Institute, University of Dundee, Dundee, United Kingdom; 2 NHS Tayside Clinical Radiology, Ninewells Hospital, Dundee, United Kingdom; 3 Vascular & Inflammatory Diseases Research Unit, Medical Research Institute, University of Dundee, Dundee, United Kingdom; 4 University of Dundee, Ninewells Hospital & Medical School, Dundee, United Kingdom; 5 NHS Tayside Medical Physics, Ninewells Hospital, Dundee, United Kingdom; 6 Dundee epidemiological and biostatistics unit, University of Dundee, Dundee, United Kingdom; INRCA, Italy

## Abstract

**Background:**

Common carotid intima media thickness (CIMT) and ankle brachial pressure index (ABPI) are used as surrogate marker of atherosclerosis, and have been shown to correlate with arterial stiffness, however their correlation with global atherosclerotic burden has not been previously assessed. We compare CIMT and ABPI with atheroma burden as measured by whole body magnetic resonance angiography (WB-MRA).

**Methods:**

50 patients with symptomatic peripheral arterial disease were recruited. CIMT was measured using ultrasound while rest and exercise ABPI were performed. WB-MRA was performed in a 1.5T MRI scanner using 4 volume acquisitions with a divided dose of intravenous gadolinium gadoterate meglumine (Dotarem, Guerbet, FR). The WB-MRA data was divided into 31 anatomical arterial segments with each scored according to degree of luminal narrowing: 0 = normal, 1 = <50%, 2 = 50–70%, 3 = 70–99%, 4 = vessel occlusion. The segment scores were summed and from this a standardized atheroma score was calculated.

**Results:**

The atherosclerotic burden was high with a standardised atheroma score of 39.5±11. Common CIMT showed a positive correlation with the whole body atheroma score (β 0.32, p = 0.045), however this was due to its strong correlation with the neck and thoracic segments (β 0.42 p = 0.01) with no correlation with the rest of the body. ABPI correlated with the whole body atheroma score (β −0.39, p = 0.012), which was due to a strong correlation with the ilio-femoral vessels with no correlation with the thoracic or neck vessels. On multiple linear regression, no correlation between CIMT and global atheroma burden was present (β 0.13 p = 0.45), while the correlation between ABPI and atheroma burden persisted (β −0.45 p = 0.005).

**Conclusion:**

ABPI but not CIMT correlates with global atheroma burden as measured by whole body contrast enhanced magnetic resonance angiography in a population with symptomatic peripheral arterial disease. However this is primarily due to a strong correlation with ilio-femoral atheroma burden.

## Introduction

Common carotid intima media thickness (CIMT), ankle brachial pressure index (ABPI) and whole body magnetic resonance angiography (WB-MRA) are all markers of atherosclerosis.

Common carotid intima media thickness is a measure of early atherosclerosis and vascular remodelling which correlates highly with standard cardiovascular risk factors. This has been acknowledged by the FDA who have approved it as a marker of atherosclerosis [1]. A reduction of CIMT thickness to lipid lowering agents has also been shown [2], [3]. These factors have led to the conclusion that it meets the criteria as a surrogate for CV disease endpoints [4], [5], and can therefore be used as a primary outcome in clinical trials to allow for more rapid assessment and development of more effective treatments at lower cost [3], [6]. However its success in predicting risk of future cardiovascular events has been mixed with a recent meta-analysis showing it to add little benefit over the Framingham risk score in predicting future cardiovascular events [1], [7]–[9]. Additionally while some studies have shown good response of CIMT with statins, the ENHANCE trial showed a rise in CIMT with statins and ezetemide despite improved biochemical markers [3], [10].

The ankle brachial pressure index has primarily been used for the diagnosis and follow-up of lower extremity arterial disease, and has been shown to correlate with future cardiovascular events in groups with and without established peripheral arterial disease [11]. Thus this has been suggested as a surrogate marker for systemic atherosclerosis [12].

Whole body contrast-enhanced magnetic resonance angiography provides visualisation of the arterial tree from the skull vertex to the pedal arteries following the intravenous injection of a gadolinium based contrast agent. It can be used to quantify atheroma burden by scoring and summating the extent of stenosis in the arterial territories of the entire body [13]. As atherosclerosis is a body wide process, an advantage of WB-MRA is that both symptomatic and asymptomatic lesions throughout the arterial tree can be detected and quantified, potentially providing more information about future vascular risk. Studies have shown WB-MRA to produce reproducible high quality images of the arterial tree [14]–[16], and that the extent of atherosclerosis demonstrated on WB-MRA correlates with traditional Framingham risk factors, severity of coronary artery disease and prediction of major adverse cardiovascular events (MACE) [13], [17]–[19].

To better understand the role of common CIMT and ABPI as markers of global atherosclerosis, it is important to know what features of atherosclerosis they accurately reflect. CIMT has been shown to correlate highly with arterial stiffness but not endothelial function [20], while ABPI has been shown to correlate with aortic stiffness and weakly with endothelial dysfunction [21], [22]. While studies have looked into the relationship between coronary angiography findings and CIMT [23], [24], and ABPI [25], [26], the correlation between these and an objective measurement of the global stenotic atheroma burden throughout the body has not been reported. Therefore we set out to assess the correlation between CIMT and ABPI and whole body atheroma burden as demonstrated on WB-MRA.

## Methodology

Ethical approval was granted by the East of Scotland Research Ethics Committee. Fifty consecutive patients with symptomatic peripheral artery disease referred for magnetic resonance angiography of their lower limbs were recruited to the study with written informed consent obtained from all participants. Disease severity was classified using the Fontaine scoring system. All patients underwent WB-MRA, ankle-brachial pressure index (ABPI), exercise tolerance testing, and CIMT measurement. These were performed on the same day, with the operators and image readers blinded to the patients' clinical status and results of the other tests.

### WB-MRA

**Figure 1:**
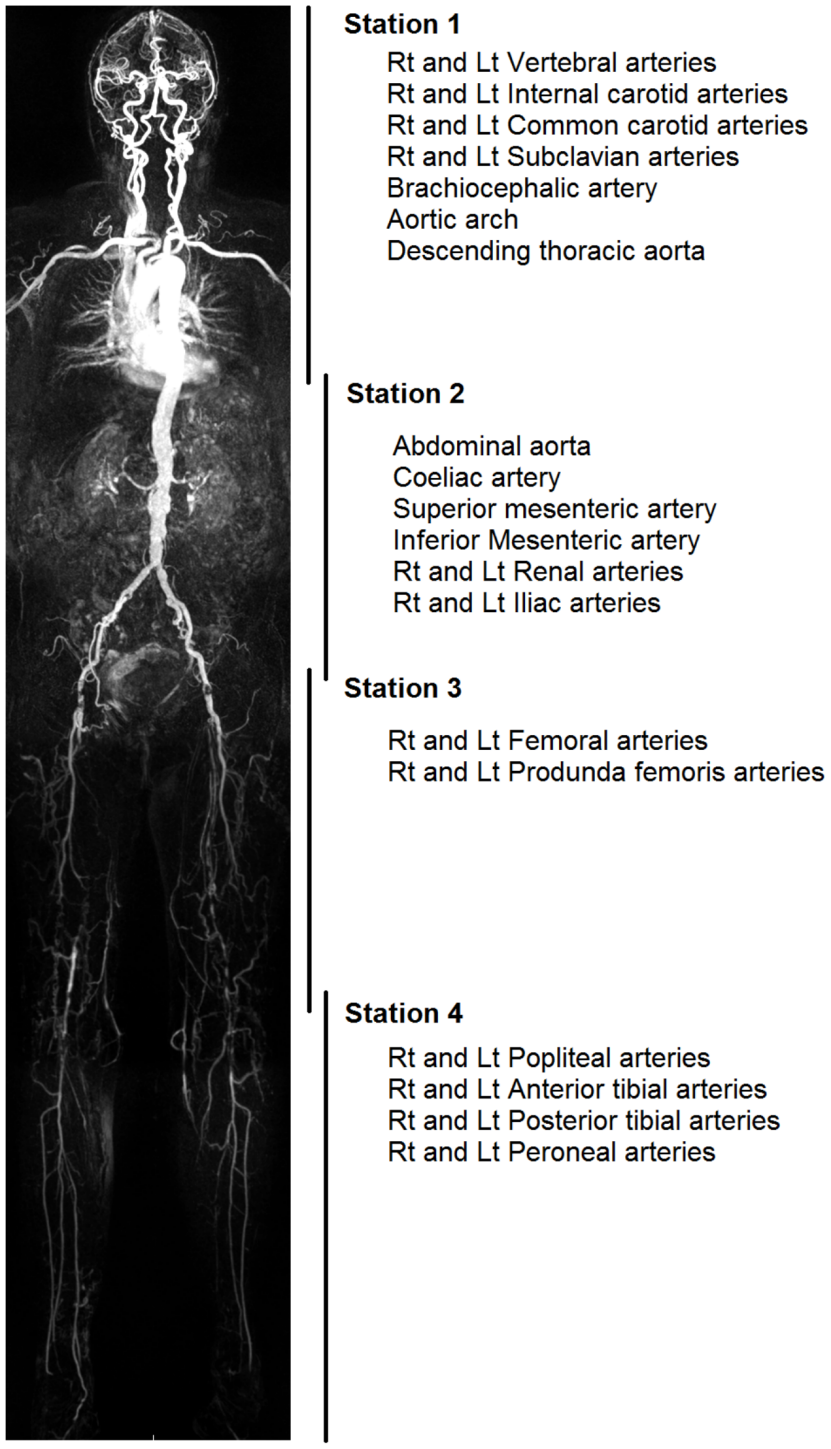
Diagram of the whole body magnetic resonance angiography stations and arterial segments. Maximum intensity projection image derived from the whole body angiogram from a typical patient in the study lies on the far left. To the right of this are the 4 overlapping 3D volume acquisitions acquired with a breakdown of the 31 arterial segments according to which station they are acquired in.

Each patient underwent whole body contrast enhanced MRI angiography using a 1.5T MRI unit (Siemens Magnetom Avanto Erlangen, Germany). Patients were imaged head first and supine in the magnet bore, with surface coils used to cover the entire body, using separate head matrix, neck matrix, spine matrix, two body matrix coils and a peripheral angiography coil. 4 stations were imaged: station 1 covered the vessels of the head and neck; station 2 the vessels of the abdomen; station 3 the upper legs; and station 4 the lower legs.(See Figure 1) Each station was acquired using a 3D TurboFLASH sequence pre and post contrast, Table 1 describes the acquisition parameters used for each of the four stations. 0.05M Intravenous gadolinium gadoterate meglumine (Dotarem, Guerbet, FR) was administered via a 20G venflon in the antecubital fossa using a two-injection contrast technique. Each injection involved a 20 ml bolus of contrast followed by 20 mls of normal saline at 1 ml/s by an injector pump. Prior to the first injection, pre-contrast images were acquired of station 1 and 4. After this the first injection was administered a Care bolus sagittal-oblique fluoroscopic image through the aortic arch was used to manually trigger image acquisition as contrast reached the aortic arch. Following acquisition of station 1, station 4 acquisition immediately began. Three sequential TurboFLASH sequences were performed of station 4 to optimise acquisition of peak contrast enhancement of the calf vessels. A 10 minute delay occurred between the end of image acquisition and the second contrast injection to allow contrast washout and minimise venous contamination [27]. After the second injection of contrast, a Care bolus coronal oblique fluoroscopic image was used to guide triggering, starting image acquisition for station 2 when contrast reached the proximal abdominal aorta. TurboFLASH sequence of station 3 was acquired immediately following this.

**Table 1:** The different sequence parameters used for the 4 stations of WB-MRA.

Parameter	Station 1	Station 2	Station 3	Station 4
Repetition Time (ms)	3.15	3.23	3.23	3.23
Echo Time (ms)	1.06	1.1	1.08	1.1
Flip angle (°)	25	25	25	25
Bandwidth (Hz/px)	450	450	450	450
Field of View	500×375	500×335	500×335	500×335
Number of slices	104	80	80	72
Matrix	384×307	384×345	384×326	384×345
Voxel size (mm^3^)	1.3×1.2×1.2	1.3×1.0×1.4	1.3×1.0×1.4	1.3×1.0×1.4
GRAPPA factor	2	2	2	2
k-space ordering	centric	linear	centric	Centric
Imaging Time (s)	18	16	16	46

The resulting images were analysed by dividing the arterial tree into 31 distinct anatomical segments. See figure 1 for a description of the stations and anatomical segments. All images were analysed using the raw data, with the aid of multiplanar reformats and maximum intensity projections on a Kodak Carestream workstation (PACS Client Suite Version 10.1 sp1, Rochester, NY, USA). Each segment was scored using a 5-point scoring system:

- 0: Normal vessels
- 1: <50% stenosis
- 2: 50–70% stenosis
- 3: >70% stenosis
- 4: Completely occluded vessel

Each segment was scored according to the maximum stenosis present at any point along the length of the vessel. Arterial segments that were not visualised with sufficient clarity for grading of the degree of stenosis were not analysed. To account for this, the final score was divided by the number of segments that had been successfully analysed to produce a normalised whole body atheroma score. The ‘standardised atheroma score’ (SAS) was calculated using equation [1]; standardisation was achieved by dividing by the maximum potential vessel score of 4.
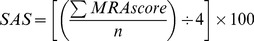
(1)


For regional atheroma assessment the arterial tree was split into 4 distinct regions with a standardised atheroma score calculated for each of these:

Thoracic – Carotids, vertebrals, subclavians, brachiocephalic and thoracic aorta;

Abdominal – Abdominal aorta, coeliac, superior and inferior mesenteric arteries and renal arteries;

Pelvic/thighs – Iliacs, femoral and femoral profundas;

Lower limb – Popliteals, anterior tibials, posterior tibials and peroneals.

### Carotid Intima Media Thickness

Ultrasound imaging of the common carotid artery (CCA) was acquired using an Acuson Sequoia with a 6 MHz Linear transducer. All scans were performed by a single operator. For the measurements the patient was lying supine on the couch. The participant was rested for at least 10 minutes in a supine position and at an environmental temperature of 25°C before any measurements were taken. A plastic arch with degrees of angulation marked on it was placed around the neck. The head was slightly extended and held in a straight position. The common carotid artery (CCA) was assessed from the clavicle to the bulb both in transverse and longitudinal section. In order to provide reproducibility the CCA was assessed at a point 1 cm central to the bulb in the longitudinal position. An image was deemed acceptable if a length of greater than 1 cm of continuous IMT could be visualised. The angle of the transducer to the skin at this point was recorded using the plastic arch. Images were recorded continuously for 10 seconds, stored in the DICOM (Digital Imaging and Communications in Medicine) file format, and subsequently analysed using the Vascular Tools Carotid Analyzer for Research software (Medical Imaging Applications LLC, Coralville, USA). The IMT of the far wall was measured using a semi-automatic technique. A user-defined region of interest was selected in a 10 mm segment of the distal wall of the common carotid artery 10 mm central to the bulb in an area without any focal plaque. The software then automatically detected the intima-media borders within this region and calculated a mean CIMT. Both carotids were measured in this manner and each side was measured twice. A mean CIMT thickness was then calculated from the four measures, as previously described [28].

### Ankle brachial pressure index

The resting pressure was taken after the patient had been supine for 10 minutes to ensure a true resting pressure. A sphygmomanometer cuff was placed around each arm in turn and each leg in turn. Arm: A continuous wave (CW) pencil probe Doppler is placed over the brachial artery in the cubital fossa. Once the signal is heard the cuff is inflated to occlude the brachial artery. It is then slowly released and the pressure at which the signal returns is recorded. This is repeated for the other arm. Legs: The cuff is place around each leg, just above the level of the malleolus. Both the Posterior Tibial Artery (PTA) and Dorsalis Pedis (DP) are insonated with the CW probe. The cuff and systolic measurements are recorded in the same manner as the brachial arteries.

The ABPI was calculated as:
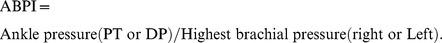



Both the American Heart Association method of using the highest of the two ankle pressures [29], and a modified technique of using the lowest ankle pressure were used [12], [30].

### Exercise treadmill test

The patient is subsequently exercised on the treadmill at a speed of 4 Kph and a gradient of 10% for one minute. After this they return immediately to the examination couch and the highest ankle pressure on each side together with the arm with the highest resting brachial pressure is re assessed. A post exercise ABPI is recorded. The lowest of the right and left post-exercise ABPI was used for analysis.

### Statistical analysis

All statistical analyses were performed with IBM SPSS Statistics for Windows, version 21.0 (IBM Corp., Armonk, NY, USA). Normality of continuous variables was tested with the Kolmogorov-Smirnov test. Where a variable failed normality testing, a standard transformation of the variable was performed using a quadratic, reciprocal or logarithmic transformation as appropriate. Data are presented as frequencies or percentages for categorical variables, as means ± SD for normally distributed continuous variables, and as median with interquartile range (IQR; 25–75^th^ percentile) for skewed variables. Correlations were performed between WB-SAS, CIMT, ABPI and population demographic metrics using the Pearson product-moment correlation coefficient. Univariate linear regression analysis was performed between the whole body atheroma score and each of the demographic variables and CIMT and ABPI. All variables with a p<0.3 were entered into a multiple linear regression with the Standardised Atheroma Score as the dependant variable and the remainder as independent variables. A p-value of <0.05 was considered significant.

## Results

### Demographic data

Fifty consecutive patients were recruited to the study. Four patients were excluded due to inability to complete the MRI examination due to claustrophobia, leaving 46 participants in the final analysis. Of these, 65% (n = 30) were male and 35% (n = 16) females, with a mean age of 64.9±10 years. 41 participants were smokers or ex smokers with a 35 (IQR 20–50) pack year history. A summary of demographic data is provided in Table 2.

**Table 2:** Demographic data of the study population.

Characteristic	Study cohort (n = 46)
Age (yrs)	64.9±10
Sex	30 male; 16 female
Systolic BP (mmHg)	146±17
Diastolic BP (mmHg)	78±12
BMI(kg/m^2^)	27.7 (IQR 25–30)
Smoking	35 (IQR 20–50)
Diabetes (n)	12
IHD (n)	18
Previous CVA (n)	10
**Medications**	
Antiplatelet	37 (80)
Statin	35 (76)
Antihypertensive agents:	33 (72)
1	12
2	13
≥3	8
Anti-claudicant	2 (4)
Anti-anginal	7 (15)

In terms of symptomatic peripheral arterial disease severity, 2 patients were Fontaine 1, 2 were Fontaine IIa, 35 were Fontaine IIb, 5 were Fontaine III and 2 were Fontaine IV. A significant proportion of patients were on medications for risk factor modification (see Table 2).

### WB-MRA

Forty-six of the fifty participants were able to complete the study. Thus there were a potential 1,426 segments for analysis. Of the 1,426 possible segments, 1395 (98%) were of sufficient quality for analysis. Of the 1,395 analysed segments, 628 were normal (45%), 346 had <50% stenosis (25%), 160 had 50–70% stenosis (12%), 169 had >70% stenosis (12%), and 77 were occluded (6%). Total atheroma burden was high, with a mean WB-SAS of 39.48±10.96.

WB-SAS increased as the clinical severity of the disease increased (see Table 3) apart from in the most severely symptomatic group.

**Table 3:** Comparison of whole body standardised atheroma score (WB-SAS) and ankle-brachial pressure index (ABPI) by Fontaine Score.

Fontaine Score	WB-SAS	ABPI resting	ABPI exercise
I	11.5±3.7	1.38±0.63	1.08 (0.33–1.16)
IIa	21.4±5.1	1.04±0.01	0.46 (0.34–0.57)
IIb	26.8±10	0.81±0.27	0.27 (0.08–0.68)
III	38.3±12.7	0.85±0.22	0.26 (0.19–0.33)
IV	19.8±2.9	1.08±0.4	0.77 (0.53–1)

WB-SAS and ABPI rest is given as mean ± SD. ABPI exercise is given as median (IQR).

### CIMT

It was not possible to obtain technically adequate images in six of the fifty patients leaving 44 for analysis. The mean common CIMTs were raised bilaterally. The CIMT was 0.98 mm (IQR 0.7–1.25) on the right and 1.02 mm (IQR 0.66–1.38) on the left. Right and left cIMT correlated highly with one another (correlation co-efficient 0.57; p<0.001). Mean CIMT was 1.00 mm (IQR 0.72–1.27). In a sub cohort of 15 individuals scanned on two different days by the same operator, the coefficient of variation of the mean CIMT was 5%.

### Ankle Brachial Pressure Index

The ABPI in the observed population was low. Using the AHA technique, the highest ABPI was 0.87±0.33, while the alternate ABPI measurement using the lower of the two ankle resulted in a median ABPI of 0.79 (IQR 0.48–1.1). On exercising the ABPI further fell to 0.52 (IQR 0.19–0.85). Table 3 documents the ABPI according to clinical severity as measured by the Fontaine classification.

### Correlation with WBAS

Common CIMT showed correlation (β 0.32 p = 0.045) with the whole body atheroma score on the univariate analysis. When this was looked at on a regional basis, CIMT only correlated with the thoracic and neck station (β 0.42 p = 0.011) with no correlation with any other anatomical region. WBAS correlated highly with age but no other demographic factor (Table 4). On multiple linear regression adjusting for confounding factors (age, systolic and diastolic blood pressure, diabetic status, and statin usage) this significance disappeared for both the whole body (β 0.13 p = 0.45) with a non-significant trend towards association with the thoracic atheroma burden (β 0.33 p = 0.07). (See table 5)

**Table 4:** Univariate linear regression analysis of variables against whole body atheroma score.

Variable	B-coefficient	p-value
Age	0.51	<0.0001
Sex	0.15	0.31
Systolic blood pressure	0.19	0.22
Diastolic blood pressure	−0.31	0.04
Diabetes	0.18	0.24
Statin therapy	0.19	0.24
BMI	0.07	0.96
Smoking status	0.05	0.77
CIMT	0.32	0.045
ABPI - rest	−0.34	0.03
ABPI – exercise	−0.48	0.003

**Table 5:** Relationships between standardised atheroma scores by region and common carotid intima media thickness (CIMT) and rest ankle-brachial pressure index (ABPI).

Region	Unadjusted model		Adjusted model*	
	CIMT	ABPI	CIMT	ABPI
Whole body	0.32‡	−0.39‡	0.13	−0.45‡
Thoracic	0.42‡	−0.17	0.33	−0.45
Abdomen	0.27	−0.30‡	0.2	−0.47‡
Ilio-femoral	0.09	−0.52‡	0.1	−0.56‡
Distal run-off	0.06	−0.13	0.19	−0.62

The ABPI values used for this analysis are the higher of the two lower limb measurements.

* Adjusted model accounts for all factors with a p>0.3 in the univariate analysis. Thus the model was adjusted for age, systolic and diastolic blood pressure, presence of diabetes and prescription of statins. Results expressed as β co-efficient.

‡ p<0.05.

ABPI significantly correlated with the whole body SAS. The AHA method of using the higher ankle pressure correlated better with the whole body SAS (β −0.39 p = 0.012) than using the lower ankle pressure (β −0.34 p = 0.03). This improved correlation continued when assessing the regional distribution, and strengthened on the multivariate analysis (β −0.45 p = 0.005 for highest ABPI, vs. β −0.31 p = 0.05 for the lowest ABPI). ABPI only correlated with the ilio-femoral vessels on regional analysis, with no correlation with any other anatomical region. Post exercise ABPI also showed significant correlation with the WBAS (β −0.48 p = 0.003) on univariate analysis, however on multivariate analysis this became insignificant (β −0.17 p = 0.17).

## Discussion

In our study we found a significant correlation between CIMT and whole body atheroma burden, however this was entirely reliant on atheroma burden within the local vessels, and this link was lost when common confounding factors were accounted for. ABPI correlated with the whole body atheroma burden, but this was mediated by a strong correlation with the iliofemoral vessels with no correlation seen with distal anatomical regions. Unlike CIMT, this correlation persisted on multivariable linear regression.

The lack of evidence of correlation between common CIMT and global atherosclerotic burden seen in our study suggests that intima media thickening may not correlate with luminal stenosis secondary to plaque formation. Given that this is the key feature of the pathophysiological process of atherosclerotic disease that guides treatment and risk stratification of coronary artery disease, cerebrovascular disease and peripheral arterial disease this lack of correlation may explain the lack of additional benefit to traditional scoring methods [9]. This correlates well with a recent study showing atherosclerotic burden as measured by WB-MRA to correlate with future major adverse cardiovascular events (MACE) in an elderly patient population (aged 70 years), with no correlation seen between CIMT and MACE [18]. In this study by Lundberg et al. addition of CIMT to the WB-MRA atheroma score improved prediction, suggesting that while CIMT is not indicative of body wide atheroma burden, it may still provide additional useful data about the underlying health of the arteries and as a marker of disease. The correlation with the thoracic atheroma burden in our study may explain the previous observation that CIMT better predicts strokes than it does coronary heart disease [31], [32]. Previous studies have shown both CIMT and WB-MRA to correlate with cardiovascular risk factors, major adverse cardiovascular events and arterial stiffness but not endothelial function, itself a risk factor for cardiovascular disease [33], thus suggesting they all represent different stages and processes in the multifaceted disorder that is atherosclerosis [20], [34], [35]. Thus CIMT may provide information on local arterial remodelling but not plaque formation, which may account for the observed improved risk stratification when added to the WBAS.

WB-MRA has been shown to accurately delineate the site and severity of peripheral arterial disease and in the current study, ABPI correlated highly with both the whole body and ilio-femoral regional atheroma score [36]. Use of the higher ABPI provided greater prediction of the atheroma burden than use of the lower ABPI. While previous studies have suggested the use of the lower threshold due to increased sensitivity for high risk patients [12], [30], our results suggest this method over diagnoses the extent of atherosclerosis. This is in support of the current recommendations from the AHA, which advise that use of the lower value leads to reduced specificity and lower risk prediction by decreasing the threshold for diagnosis of significant atherosclerotic disease [29]. The correlation observed in our population between ABPI and WBAS is in keeping with that observed by Lehrke et al. in patients with coronary artery disease but without peripheral arterial disease [17].

The correlation of CIMT and ABPI with local stenotic disease but not with disease in other areas of the body suggests a complimentary role for these two techniques in the assessment of the arterial tree. Indeed the Rotterdam study showed improvement in the risk stratification from the combination of these techniques [37]. Further work is required to assess the complimentary or exclusive role of WB-MRA with CIMT and ABPI in risk stratification and prediction.

Limitations of the study include the patient selection, which examined patients with symptomatic peripheral vascular disease, a group at a very advanced stage of the cardiovascular disease spectrum. Further work is thus warranted to look at the ability of CIMT and ABPI to predict atheroma burden in a healthier population. The population was predominantly Fontaine type IIb patients, which is not surprising as these are the patients most likely to be considered for intervention, and therefore for vascular imaging, however this group may have skewed results. 78% of the participants were on statin therapy. Some studies have shown CIMT to improve with statin therapy [3], and that statins have been shown to replace the fibrofatty component of plaques with calcific plaque without reducing plaque volume [38]. Given that plaque volume is the key contributor to the degree of stenosis used to calculate the WBAS, this may have confounded the results. However, a separate study by groups have found no reduction in CIMT with statin therapy [10], and a study using MRI volumetric plaque analysis showed statins to reduce plaque volume in the carotid arteries despite no change in the CIMT [39]. Thus the impact of statin therapy on the current results is uncertain.

In the current study we used common CIMT as this is the most established technique for assessing IMT, however the internal CIMT and carotid plaque dimensions on ultrasound have both been suggested as more discerning alternatives to common CIMT [40], [41].

Additionally WB-MRA does not assess the coronary vessels. Coronary MRA is still someway off routine clinical practice although this is a promising area with several studies showing accurate assessment of the proximal vessels and integration of this in the future is a possibility [42].

In conclusion, CIMT and ABPI are markers of local atherosclerotic stenotic plaque burden but not systemic plaque burden in a population with peripheral arterial disease. Thus the techniques may be complimentary in their assessment of the vascular system.

## References

[1] Lorenz MW, Schaefer C, Steinmetz H, Sitzer M (2010) Is carotid intima media thickness useful for individual prediction of cardiovascular risk? Ten-year results from the Carotid Atherosclerosis Progression Study (CAPS). Eur Heart J 31: 2041–2048 Available: http://www.ncbi.nlm.nih.gov/pubmed/20530503. Accessed 2013 Dec 29. 20530503 10.1093/eurheartj/ehq189

[2] Crouse JR, Raichlen JS, Riley WA, Evans GW, Palmer MK, et al (2007) Effect of rosuvastatin on progression of carotid intima-media thickness in low-risk individuals with subclinical atherosclerosis: the METEOR Trial. JAMA 297: 1344–1353 Available: http://www.ncbi.nlm.nih.gov/pubmed/17384434. Accessed 2013 Dec 29. 17384434 10.1001/jama.297.12.1344

[3] Espeland MA, O'leary DH, Terry JG, Morgan T, Evans G, et al (2005) Carotid intimal-media thickness as a surrogate for cardiovascular disease events in trials of HMG-CoA reductase inhibitors. Curr Control Trials Cardiovasc Med 6: 3 Available: http://www.pubmedcentral.nih.gov/articlerender.fcgi?artid=555546&tool=pmcentrez&rendertype=abstract. Accessed 2013 Dec 29. 15760471 10.1186/1468-6708-6-3 PMC555546

[4] Fitch KV, Stavrou E, Looby SE, Hemphill L, Jaff MR, et al (2011) Associations of cardiovascular risk factors with two surrogate markers of subclinical atherosclerosis: endothelial function and carotid intima media thickness. Atherosclerosis 217: 437–440 Available: http://www.pubmedcentral.nih.gov/articlerender.fcgi?artid=3146552&tool=pmcentrez&rendertype=abstract. Accessed 2013 Dec 29. 21570076 10.1016/j.atherosclerosis.2011.04.009 PMC3146552

[5] Peters SAE, Grobbee DE, Bots ML (2011) Carotid intima–media thickness: a suitable alternative for cardiovascular risk as outcome? Eur J Cardiovasc Prev Rehabil 18: 167–174 Available: http://www.ncbi.nlm.nih.gov/pubmed/21568017. Accessed 2013 Dec 29. 21568017 10.1177/1741826710389400

[6] Bots ML, Evans GW, Riley WA, Grobbee DE (2003) Carotid intima-media thickness measurements in intervention studies: design options, progression rates, and sample size considerations: a point of view. Stroke 34: 2985–2994 Available: http://www.ncbi.nlm.nih.gov/pubmed/14615619. Accessed 2013 Dec 29. 14615619 10.1161/01.STR.0000102044.27905.B5

[7] Costanzo P, Perrone-Filardi P, Vassallo E, Paolillo S, Cesarano P, et al (2010) Does carotid intima-media thickness regression predict reduction of cardiovascular events? A meta-analysis of 41 randomized trials. J Am Coll Cardiol 56: 2006–2020 Available: http://www.ncbi.nlm.nih.gov/pubmed/21126642. Accessed 2013 Dec 29. 21126642 10.1016/j.jacc.2010.05.059

[8] Helfand M, Buckley DI, Freeman M, Fu R, Rogers K, et al (2009) Emerging risk factors for coronary heart disease: a summary of systematic reviews conducted for the U.S. Preventive Services Task Force. Ann Intern Med 151: 496–507 Available: http://www.ncbi.nlm.nih.gov/pubmed/19805772. Accessed 2013 Dec 13. 19805772 10.7326/0003-4819-151-7-200910060-00010

[9] Den Ruijter HM, Peters SaE, Anderson TJ, Britton AR, Dekker JM, et al (2012) Common carotid intima-media thickness measurements in cardiovascular risk prediction: a meta-analysis. JAMA 308: 796–803 Available: http://www.ncbi.nlm.nih.gov/pubmed/22999719. Accessed 2013 Dec 18. 22910757 10.1001/jama.2012.9630

[10] Kastelein JJP, Akdim F, Stroes ESG, Zwinderman AH, Bots ML, et al (2008) Simvastatin with or without ezetimibe in familial hypercholesterolemia. N Engl J Med 358: 1431–1443 Available: http://www.ncbi.nlm.nih.gov/pubmed/18376000. Accessed 2013 Dec 30. 18376000 10.1056/NEJMoa0800742

[11] Fowkes FGR, Murray GD, Butcher I, Heald CL, Lee RJ, et al (2008) Ankle brachial index combined with Framingham Risk Score to predict cardiovascular events and mortality: a meta-analysis. JAMA 300: 197–208 Available: http://www.pubmedcentral.nih.gov/articlerender.fcgi?artid=2932628&tool=pmcentrez&rendertype=abstract. Accessed 2014 Feb 2. 18612117 10.1001/jama.300.2.197 PMC2932628

[12] Espinola-Klein C, Rupprecht HJ, Bickel C, Lackner K, Savvidis S, et al (2008) Different calculations of ankle-brachial index and their impact on cardiovascular risk prediction. Circulation 118: 961–967 Available: http://www.ncbi.nlm.nih.gov/pubmed/18697822. Accessed 2014 Feb 2. 18697822 10.1161/CIRCULATIONAHA.107.763227

[13] Hansen T, Ahlström H, Wikström J, Lind L, Johansson L (2008) A total atherosclerotic score for whole-body MRA and its relation to traditional cardiovascular risk factors. Eur Radiol 18: 1174–1180 Available: http://www.ncbi.nlm.nih.gov/pubmed/18270716. Accessed 2014 Feb 4. 18270716 10.1007/s00330-008-0864-6

[14] Fenchel M, Scheule AM, Stauder NI, Kramer U, Tomaschko K, et al (2006) Atherosclerotic disease: whole-body cardiovascular imaging with MR system with 32 receiver channels and total-body surface coil technology–initial clinical results. Radiology 238: 280–291 Available: http://www.ncbi.nlm.nih.gov/pubmed/16304083. Accessed 2013 Dec 29. 16304083 10.1148/radiol.2381041532

[15] Ruehm SG, Goehde SC, Goyen M (2004) Whole body MR angiography screening. Int J Cardiovasc Imaging 20: 587–591 Available: http://www.ncbi.nlm.nih.gov/pubmed/15856646. Accessed 2013 Dec 29. 15856646 10.1007/s10554-004-7033-z

[16] Goehde SC, Hunold P, Vogt FM, Ajaj W, Goyen M, et al (2005) Full-body cardiovascular and tumor MRI for early detection of disease: feasibility and initial experience in 298 subjects. AJR Am J Roentgenol 184: 598–611 Available: http://www.ajronline.org/doi/abs/10.2214/ajr.184.2.01840598. Accessed 2013 Dec 19. 15671386 10.2214/ajr.184.2.01840598

[17] Lehrke S, Egenlauf B, Steen H, Lossnitzer D, Korosoglou G, et al (2009) Prediction of coronary artery disease by a systemic atherosclerosis score index derived from whole-body MR angiography. J Cardiovasc Magn Reson 11: 36 Available: http://www.pubmedcentral.nih.gov/articlerender.fcgi?artid=2758875&tool=pmcentrez&rendertype=abstract. Accessed 2013 Dec 19. 19761595 10.1186/1532-429X-11-36 PMC2758875

[18] Lundberg C, Johansson L, Barbier CE, Lind L, Ahlström H, et al (2013) Total atherosclerotic burden by whole body magnetic resonance angiography predicts major adverse cardiovascular events. Atherosclerosis 228: 148–152 Available: http://www.sciencedirect.com/science/article/pii/S0021915013001251. Accessed 2013 Dec 19. 23474127 10.1016/j.atherosclerosis.2013.02.015

[19] Bamberg F, Parhofer KG, Lochner E, Marcus RP, Theisen D, et al (2013) Diabetes Mellitus: Long-term Prognostic Value of Whole-Body MR Imaging for the Occurrence of Cardiac and Cerebrovascular Events. Radiology 269: 730–737 Available: http://www.ncbi.nlm.nih.gov/pubmed/24023074. Accessed 2013 Dec 19. 24023074 10.1148/radiol.13130371

[20] Lind L, Andersson J, Hansen T, Johansson L, Ahlström H (2009) Atherosclerosis measured by whole body magnetic resonance angiography and carotid artery ultrasound is related to arterial compliance, but not to endothelium-dependent vasodilation - the Prospective Investigation of the Vasculature in Uppsala Seniors (PIV. Clin Physiol Funct Imaging 29: 321–329 Available: http://www.ncbi.nlm.nih.gov/pubmed/19486081. Accessed 2013 Jun 14. 19486081 10.1111/j.1475-097X.2009.00871.x

[21] Matsumae T, Abe Y, Murakami G, Ishihara M, Ueda K, et al (2007) Determinants of arterial wall stiffness and peripheral artery occlusive disease in nondiabetic hemodialysis patients. Hypertens Res 30: 377–385 Available: http://www.ncbi.nlm.nih.gov/pubmed/17587749. Accessed 2014 Apr 18. 17587749 10.1291/hypres.30.377

[22] Brevetti G, Silvestro A, Schiano V, Chiariello M (2003) Endothelial dysfunction and cardiovascular risk prediction in peripheral arterial disease: additive value of flow-mediated dilation to ankle-brachial pressure index. Circulation 108: 2093–2098 Available: http://www.ncbi.nlm.nih.gov/pubmed/14530195. Accessed 2014 Apr 18. 14530195 10.1161/01.CIR.0000095273.92468.D9

[23] Coskun U, Yildiz A, Esen OB, Baskurt M, Cakar MA, et al (2009) Relationship between carotid intima-media thickness and coronary angiographic findings: a prospective study. doi:10.1186/1476-7120-7-59 10.1186/1476-7120-7-59 PMC2809045 20043836

[24] Kwon T-G, Kim K-W, Park H-W, Jeong J-H, Kim K-Y, et al (2009) Prevalence and significance of carotid plaques in patients with coronary atherosclerosis. Korean Circ J 39: 317–321 doi:10.4070/kcj.2009.39.8.317 19949637 10.4070/kcj.2009.39.8.317 PMC2771847

[25] Sadeghi M, Heidari R, Mostanfar B, Tavassoli A, Roghani F, et al (2011) The Relation Between Ankle-Brachial Index (ABI) and Coronary Artery Disease Severity and Risk Factors: An Angiographic Study. ARYA Atheroscler 7: 68–73 Available: http://www.pubmedcentral.nih.gov/articlerender.fcgi?artid=3347847&tool=pmcentrez&rendertype=abstract. Accessed 2014 Feb 7. 22577449 PMC3347847

[26] Papamichael CM, Lekakis JP, Stamatelopoulos KS, Papaioannou TG, Alevizaki MK, et al (2000) Ankle-brachial index as a predictor of the extent of coronary atherosclerosis and cardiovascular events in patients with coronary artery disease. Am J Cardiol 86: 615–618 Available: http://www.ncbi.nlm.nih.gov/pubmed/10980210. Accessed 2014 Jan 31. 10980210 10.1016/s0002-9149(00)01038-9

[27] Waugh SA, Ramkumar PG, Gandy SJ, Nicholas RS, Martin P, et al (2009) Optimization of the contrast dose and injection rates in whole-body MR angiography at 3.0T. J Magn Reson Imaging 30: 1059–1067 Available: http://www.ncbi.nlm.nih.gov/pubmed/19856438. Accessed 2013 Dec 19. 19856438 10.1002/jmri.21930

[28] Engelen L, Ferreira I, Stehouwer CD, Boutouyrie P, Laurent S (2013) Reference intervals for common carotid intima-media thickness measured with echotracking: relation with risk factors. Eur Heart J 34: 2368–2380 Available: http://www.ncbi.nlm.nih.gov/pubmed/23186808. Accessed 2014 Apr 17. 23186808 10.1093/eurheartj/ehs380

[29] Aboyans V, Criqui MH, Abraham P, Allison Ma, Creager Ma, et al (2012) Measurement and interpretation of the ankle-brachial index: a scientific statement from the American Heart Association. Circulation 126: 2890–2909 Available: http://www.ncbi.nlm.nih.gov/pubmed/23159553. Accessed 2014 Feb 3. 23159553 10.1161/CIR.0b013e318276fbcb

[30] Nead KT, Cooke JP, Olin JW, Leeper NJ (2013) Alternative ankle-brachial index method identifies additional at-risk individuals. J Am Coll Cardiol 62: 553–559 Available: http://www.ncbi.nlm.nih.gov/pubmed/23707317. Accessed 2014 Feb 2. 23707317 10.1016/j.jacc.2013.04.061 PMC3732795

[31] Lorenz MW, Markus HS, Bots ML, Rosvall M, Sitzer M (2007) Prediction of clinical cardiovascular events with carotid intima-media thickness: a systematic review and meta-analysis. Circulation 115: 459–467 Available: http://www.ncbi.nlm.nih.gov/pubmed/17242284. Accessed 2014 Apr 23. 17242284 10.1161/CIRCULATIONAHA.106.628875

[32] Folsom AR, Kronmal RA, Detrano RC, O'Leary DH, Bild DE, et al (2008) Coronary artery calcification compared with carotid intima-media thickness in the prediction of cardiovascular disease incidence: the Multi-Ethnic Study of Atherosclerosis (MESA). Arch Intern Med 168: 1333–1339 Available: http://www.pubmedcentral.nih.gov/articlerender.fcgi?artid=2555989&tool=pmcentrez&rendertype=abstract. Accessed 2014 Apr 23. 18574091 10.1001/archinte.168.12.1333 PMC2555989

[33] Lerman A, Zeiher AM (2005) Endothelial function: cardiac events. Circulation 111: 363–368 Available: http://www.ncbi.nlm.nih.gov/pubmed/15668353. Accessed 2013 Dec 31. 15668353 10.1161/01.CIR.0000153339.27064.14

[34] De Simone G, McClelland R, Gottdiener JS, Celentano A, Kronmal RA, et al (2001) Relation of hemodynamics and risk factors to ventricular-vascular interactions in the elderly: the Cardiovascular Health Study. J Hypertens 19: 1893–1903 Available: http://www.ncbi.nlm.nih.gov/pubmed/11593112. Accessed 2014 Apr 18. 11593112 10.1097/00004872-200110000-00026

[35] Yeboah J, Burke GL, Crouse JR, Herrington DM (2008) Relationship between brachial flow-mediated dilation and carotid intima-media thickness in an elderly cohort: the Cardiovascular Health Study. Atherosclerosis 197: 840–845 Available: http://www.ncbi.nlm.nih.gov/pubmed/17804000. Accessed 2014 Apr 18. 17804000 10.1016/j.atherosclerosis.2007.07.032 PMC4115586

[36] Wikström J, Hansen T, Johansson L, Ahlström H, Lind L (2009) Lower extremity artery stenosis distribution in an unselected elderly population and its relation to a reduced ankle-brachial index. J Vasc Surg 50: 330–334 Available: http://www.ncbi.nlm.nih.gov/pubmed/19446989. Accessed 2013 Dec 30. 19446989 10.1016/j.jvs.2009.03.008

[37] Van der Meer IM, Bots ML, Hofman A, del Sol AI, van der Kuip DaM, et al (2004) Predictive value of noninvasive measures of atherosclerosis for incident myocardial infarction: the Rotterdam Study. Circulation 109: 1089–1094 Available: http://www.ncbi.nlm.nih.gov/pubmed/14993130. Accessed 2014 Apr 23. 14993130 10.1161/01.CIR.0000120708.59903.1B

[38] Nozue T, Yamamoto S, Tohyama S, Umezawa S, Kunishima T, et al (2012) Statin treatment for coronary artery plaque composition based on intravascular ultrasound radiofrequency data analysis. Am Heart J 163: 191–9.e1 Available: http://www.ncbi.nlm.nih.gov/pubmed/22305836. Accessed 2013 Dec 30. 22305836 10.1016/j.ahj.2011.11.004

[39] Migrino RQ, Bowers M, Harmann L, Prost R, LaDisa JF (2011) Carotid plaque regression following 6-month statin therapy assessed by 3T cardiovascular magnetic resonance: comparison with ultrasound intima media thickness. J Cardiovasc Magn Reson 13: 37 Available: http://www.pubmedcentral.nih.gov/articlerender.fcgi?artid=3166901&tool=pmcentrez&rendertype=abstract. Accessed 2013 Dec 19. 21812992 10.1186/1532-429X-13-37 PMC3166901

[40] Polak JF, Pencina MJ, Pencina KM, O'Donnell CJ, Wolf PA, et al (2011) Carotid-wall intima-media thickness and cardiovascular events. N Engl J Med 365: 213–221 Available: http://www.pubmedcentral.nih.gov/articlerender.fcgi?artid=3153949&tool=pmcentrez&rendertype=abstract. Accessed 2013 Dec 18. 21774709 10.1056/NEJMoa1012592 PMC3153949

[41] Inaba Y, Chen JA, Bergmann SR (2012) Carotid plaque, compared with carotid intima-media thickness, more accurately predicts coronary artery disease events: a meta-analysis. Atherosclerosis 220: 128–133 Available: http://www.ncbi.nlm.nih.gov/pubmed/21764060. Accessed 2013 Dec 30. 21764060 10.1016/j.atherosclerosis.2011.06.044

[42] Sakuma H (2011) Coronary CT versus MR angiography: the role of MR angiography. Radiology 258: 340–349 Available: http://www.scopus.com/inward/record.url?eid=2-s2.0-79952398815&partnerID=40&md5=d8e12db65df5edd617bfaa06afd7e11e. Accessed 2013 Dec 30. 21273518 10.1148/radiol.10100116

